# Alertness Modulates Conflict Adaptation and Feature Integration in an Opposite Way

**DOI:** 10.1371/journal.pone.0079146

**Published:** 2013-11-08

**Authors:** Peiduo Liu, Wenjing Yang, Jia Chen, Xiting Huang, Antao Chen

**Affiliations:** 1 Key Laboratory of Cognition and Personality of Ministry of Education, Faculty of Psychology, Southwest University, Chongqing, China; 2 School of Psychology, Liaoning Normal University, Dalian, Liaoning, China; Radboud University Nijmegen, The Netherlands

## Abstract

Previous studies show that the congruency sequence effect can result from both the conflict adaptation effect (CAE) and feature integration effect which can be observed as the repetition priming effect (RPE) and feature overlap effect (FOE) depending on different experimental conditions. Evidence from neuroimaging studies suggests that a close correlation exists between the neural mechanisms of alertness-related modulations and the congruency sequence effect. However, little is known about whether and how alertness mediates the congruency sequence effect. In Experiment 1, the Attentional Networks Test (ANT) and a modified flanker task were used to evaluate whether the alertness of the attentional functions had a correlation with the CAE and RPE. In Experimental 2, the ANT and another modified flanker task were used to investigate whether alertness of the attentional functions correlate with the CAE and FOE. In Experiment 1, through the correlative analysis, we found a significant positive correlation between alertness and the CAE, and a negative correlation between the alertness and the RPE. Moreover, a significant negative correlation existed between CAE and RPE. In Experiment 2, we found a marginally significant negative correlation between the CAE and the RPE, but the correlation between alertness and FOE, CAE and FOE was not significant. These results suggest that alertness can modulate conflict adaptation and feature integration in an opposite way. Participants at the high alerting level group may tend to use the top-down cognitive processing strategy, whereas participants at the low alerting level group tend to use the bottom-up processing strategy.

## Introduction

The congruency sequence effect refers to the congruency effects (incongruent minus congruent) that are smaller, following an incongruent rather than a congruent stimulus [1]. Typically, performances (usually, reaction times (RTs)) in incongruent trials that follow incongruent trials (iI) are better (faster) than performances in those that follow congruent trials (cI). Similarly, performances in congruent trials that follow congruent trials (cC) are better than performances in those that follow incongruent trials (iC). Performance interactions between previous trial type (c, i) and current trial type (C, I) indicate the presence of the congruency sequence effect. The size of the effect can be computed as follows: RT_[(cI - cC) - (iI - iC)]_ [2].

Two main theories can be used to account for this effect. The conflict monitoring theory states that conflict detection of a preceding trial increases cognitive control, which reduces the congruency effect on the current trial [3]–[5]. The feature integration theory attributes the congruency sequence effect to an unbalanced proportion of complete or partial repetitions of stimulus-response (S-R) features [2], [6], [7]. In recent studies, there has been a debate on whether the congruency sequence effect is reflected in a conflict adaptation effect (CAE) or a feature integration effect [2], [5], [7]. CAE is considered to reflect a top-down regulation in the cognitive process based on change trials where stimuli and responses are different from the immediately preceding trials, and feature integration effect is based on episodic memory effects of S–R associations and does not invoke cognitive control mechanisms [8]. Most studies suggest that these two mechanisms seem to be involved in varying degrees, depending strongly on task context, experimental setting, and amount of feature transitions. That is, the congruency sequence effect can result from both CAE and feature integration effect [8]–[14].

Alertness is specified as phasic alertness (task specific), which is distinguished from intrinsic alertness (a general cognitive control of arousal). This capacity, which is the ability to increase and maintain response readiness in preparation for an impending stimulus, can be thought of as a foundational form of attention on which other attentional functions rest [15], [16]. Neuroimaging data suggest that the anterior cingulate cortex (ACC) is involved in both alertness-related modulation [17] and conflict monitoring [18]. On the one hand, the ACC is involved in alertness-related modulation, which arises from brain stem monoamine nuclei inputs, such as those from the mesocortical dopamine system originating in the ventral tegmental area, and norepinephrine inputs from the locus coeruleus (LC) [17], [19], [20].

On the other hand, the ACC is believed to act as a conflict monitor, which contributes to cognition by detecting the conflict occurrence during information processing, and by alerting the cognitive control system involved in top-down control to resolve the conflict [21]–[25]. Verguts & Notebaert [26] proposes that the ACC may exert its effects on dorsal lateral prefrontal cortex (DLPFC) through LC-related processing in the congruency sequence effect. Accordingly, evidence from neuroimaging studies suggests that a close correlation exists between the neural mechanisms of alertness-related modulations and the congruency sequence effect. Based on these findings, we hypothesized that the alertness may have an influence on the congruency sequence effect. In this study, we focused on the question that whether and how alertness mediates the congruency sequence effect.

We used two tasks to investigate the above issues. In Task 1, we evaluated the individual differences in the alerting level through the Attentional Networks Test (ANT)[16]. The ANT was developed to measure the efficiency of the attentional networks, which carry out the distinct functions of alerting, orienting, and executive attention[16], [27], [28]. The ANT can provide the measures on the individual differences of the alerting efficiency, which can, in turn, be used to explore the cause of the congruency sequence effect. Meanwhile, we can also investigate whether orienting and/or executive attention influence the congruency sequence effect. In Task 2, we used a modified flanker task, in which the letters N, H, O, and Q were used to form the stimuli arrays. The letters N and H have angular features, which are obviously different from the letters O and Q, which have rounded features [29]. Each letter was mapped onto one key, so there were four responses and a large stimulus and response set. As a result of the large stimulus and response set, we can establish S-R mappings to avoid the possibility of the target letters with similar features in the sequential trials being mapped onto the same key. This design allowed us to evaluate both feature integration effect and CAE in different conditions.

More importantly, the congruence sequence effect is more complicated than what is mentioned above. The congruency sequence effect can result from both CAE and feature integration effect [8]–[13]. In the meantime, the feature integration effect can be observed as the repetition priming effect (RPE) or the feature overlap effect (FOE) depending on different experimental conditions [2], [5], [7], [8], [10]. The RPE is the bottom-up feature priming process based on repetition trials where responses or stimuli-responses mappings are the same as the immediately preceding trials and it constitutes a special case of feature integration effect [2], [5], [7], [8]. And the FOE is calculated on the four different types of iI trials (ie., complete repetition iI trials, only flanker repetition iI trials, only target repetition iI trials, and complete change iI trials) in the four-choice task[10].

To further explore the relation between alertness and congruency sequence effect, it is necessary to take the different feature integration effect (that is, RPE and FOE) into consideration. For this reason, we ran two experiments and each experiment contained the two tasks mentioned above. In Experiment 1, we investigated how the alertness mediates CAE and RPE. In Experiment 2, we examined the role of alertness in CAE and FOE. Therefore, the ANT tasks in the two experiments were identical, and the difference between the two experiments was the flanker tasks in the two experiments. In Experiment 1, due to the confusion that the congruency sequence effect may be masked by negative priming, which means that RTs are unusually long for incompatible flanker stimuli when the locations of target and flanker items are reversed from those of the preceding trial (e.g., HHNHH to NNHNN) [5], they have to be removed from the task. For better controlling the negative priming effect on change trials and on response or S-R repetition trials, we used just four types of incongruent trials (see the section of methods and the Table S1). In Experiment 2, in order to calculate the FOE, all the twelve different types of incongruent trials and more trial numbers in the four-choice flanker task were created. Furthermore, all other aspects of the flanker tasks in the two experiments were the same. These designs allowed us to explore the relations between alertness and congruency sequence effect in different conditions (that is, the role of alertness in CAE and RPE, and/or in CAE and FOE). Moreover, the results from two experiments can be compared to examine the reliability of the possible novel findings.

## Experiment 1

### Materials and Methods

#### Ethics Statement

Approval of the study was made by the Human Research Ethics Committee of the Southwest University of China, and all participants provided written informed consent.

#### Participants

A total of 112 right-handed undergraduate and graduate students (62 females; mean age  = 22 years, range  = 18–26) were paid for their participation. All participants had normal or corrected-to-normal vision.

#### Stimuli and Task

The ANT followed its standard procedure (http://www.sacklerinstitute.org/users/jin.fan/). At the beginning of each trial, a fixation point was presented at a random duration of 400 – 1600 ms. Then, a cue appeared for 100 ms. There were four cue conditions: no-cue, center-cue, double-cue and spatial-cue. In the no-cue condition, only the fixation cross was presented for a variable duration from 350 ms to 650 ms. In the center-cue condition, the cue asterisk was presented at the location of the fixation cross for 100 ms. In the double-cue condition, the two asterisks were presented simultaneously at two possible target positions for 100 ms. In the spatial-cue condition, the cue asterisk was presented at the target position for 100 ms. After the cue there was a short fixation for 400 ms and then the target appeared at a visual angel of 0.96° above or below the fixation point. Target location was always uncertain except in the spatial cue trials. Participants were instructed to focus on the centrally located fixation cross throughout the task. Stimuli consisted of five arrows. When the stimulus appeared, the participants were instructed to respond as quickly and accurately as possible to the central target, specifically, they needed to press the left mouse button with left thumb if the central arrow points to the left or press the right mouse button with right thumb if the central arrow points to the right. The stimuli were presented until the subjects responded, but for no longer than 1,700 ms. After subjects made a response, the stimuli disappeared immediately and a posttarget fixation point was displayed at a variable duration (3,500 ms minus duration of the first fixation minus RT). Each subject completed 24 full-feedback practice trials before formal tests. During the formal tests, subjects performed 96 trials (4 cue conditions x 2 target locations x 2 flanker conditions x 2 central letters x 3 repetitions) for the ANT.

The flanker task on Personal Computers, using E-Prime software (Psychological Software Tools, Pittsburgh, PA), was presented. We used four different letters (two angular letters: N and H; two round letters: O and Q), which were mapped on four different responses (left middle finger, left index finger, right index finger, and right middle finger), respectively. Specifically, N was mapped onto the left middle finger (D key), O was mapped onto the left index finger (F key), Q was mapped onto the right index finger (J key), and H was mapped onto the right middle finger (K key). Participants were instructed to press the key corresponding to the central letter quickly and accurately. On each trial, a line of five letters was presented, the central one of which was the target, and the remaining letters were the flankers. On the congruent trials, the flankers were identical to the target (NNNNN, HHHHH, OOOOO, and QQQQQ). On the incongruent trials, the flankers were mapped onto the response hand opposite to the target stimulus (HHNHH, NNHNN, OOQOO, and QQOQQ). Therefore, such a design ensured that the visual features between two complete change trials were always different. All participants were asked to perform the task 1 first and then task 2.

#### Design and Procedure

The flanker experiment employed a 2×2 within-subject design, with previous trial types (c, i) and current trial types (C, I) as the factors. Each of the congruent and incongruent trials accounted for 50% of the trials. First, we put 48 trial sequences of all the four trial sequence types (cC, cI, iC, and iI, see the Table S1) in a array. Then, we pseudo-randomly generated five lists and each list contained 96 trial sequences which used 2 arrays. The last trial from the previous trial sequence was same as the first trial in the following trial sequence (the first trial of each list was excluded from the analyses), so the final list of each block was 97 trials long. Participants received 33 practice trials before entering the experimental phase, which consisted of five blocks of 97 trials each. In terms of repetition/change, all transitions (congruency of trial n-1× congruency of trial n) can be classified into three categories, namely, partial repetition (e.g., HHNHH to NNHNN), complete repetition (e.g., OOQOO to OOQOO), and complete change (e.g., HHNHH to OOQOO) (see the Table S1).

In the flanker task, the arrays were presented in the center of a computer display approximately 60 cm from participants. In each trial, a fixation was first presented 0.5° above the center of the screen for 300 ms. Then a stimuli array was presented in the center of the screen for 200 ms, followed by a blank screen for 1000 ms. When the blank screen appeared, participants were required to press the arranged key. Finally, following another 1000 ms blank screen, the next trial commenced.

### Results

First, following the standard algorithm of Fan et al.[16], three attentional functions were calculated. The alerting effect was calculated by subtracting the mean RT of the double-cue conditions from the mean RT of the no-cue conditions, with higher scores suggesting larger alerting effects due to the presentation of cues warning the participants of the upcoming target. The orienting effect was calculated by subtracting the mean RT of the spatial cue conditions from the mean RT of the center cue, with higher scores suggesting larger orienting effects based on the provision of exact spatial predictive information. The executive control effect was calculated by subtracting the mean RT of all congruent flanking conditions, summed across cue types, from the mean RT of incongruent flanking condition, with higher scores suggesting larger conflict interference and less efficiency. To evaluate the independence of attentional networks, we submitted all data to Pearson Correlation and bootstrapping correlation confidence interval analysis among the three network effects. Results showed that the efficiencies of these three networks were uncorrelated (Table 1). Inasmuch as they had no significant correlation with one another, the scores of the three networks were used for further analysis.

**Table 1:** Correlations between the related factors in Experiment 1.

			Alerting	Orienting	EC	CAE	RPE
	Pearson Correlation		.092	—			
**Orienting**	*Bootstrap^c^*	lower	−.102				
	*95% CI*	upper	.274				
	Pearson Correlation		.059	−.175	—		
**EC**	*Bootstrap^c^*	lower	−.122	−.343			
	*95% CI*	upper	.242	.001			
	Pearson Correlation		.369**	.139	.003	—	
**CAE**	*Bootstrap^c^*	lower	.214	−.038	−.212		
	*95% CI*	upper	.506	.314	.206		
	Pearson Correlation		−.230*	.024	−.092	−.265**	—
**RPE**	*Bootstrap^c^*	lower	−.385	−.172	−.268	−.483	
	*95% CI*	upper	−.017	.215	.096	−.012	
	Pearson Correlation		.010	−.107	.257**	.065	.030
**Conflict**	*Bootstrap^c^*	lower	−.193	−.298	.020	−.121	−.152
	*95% CI*	upper	.202	.072	.471	.240	.216

** . Correlation is significant at the .01 level (2-tailed).

* . Correlation is significant at the .05 level (2-tailed).

c. Unless otherwise noted, bootstrap results are based on 5000 bootstrap samples

NB. EC  =  executive control; CAE  =  conflict adaptation effect on change trials; RPE  =  repetition priming effect on the response or S-R repetition trials; and conflict  =  congruency effects in the flanker task; CI  =  Confidence Interval, respectively.

Second, in the flanker task, the first trial of each block, the trials with incorrect responses or with RTs more than 3 SDs from the mean RTs of each participant, and the trials immediately following incorrect trials were excluded from RT analyses (6.9% of all the trials). The preliminary analysis was based on the RT_[(cI - cC) - (iI - iC)]_, which was used to describe the CAE [2] and was useful for studying group differences in the cognitive control [30]–[32]. We noted the RT_[(cI - cC) - (iI - iC)]_ for the change trials, which usually indexes the CAE, and the RT_[(cI - cC) - (iI - iC)]_ for the response or S-R repetition trials, which indexes the RPE.

**Figure 1:**
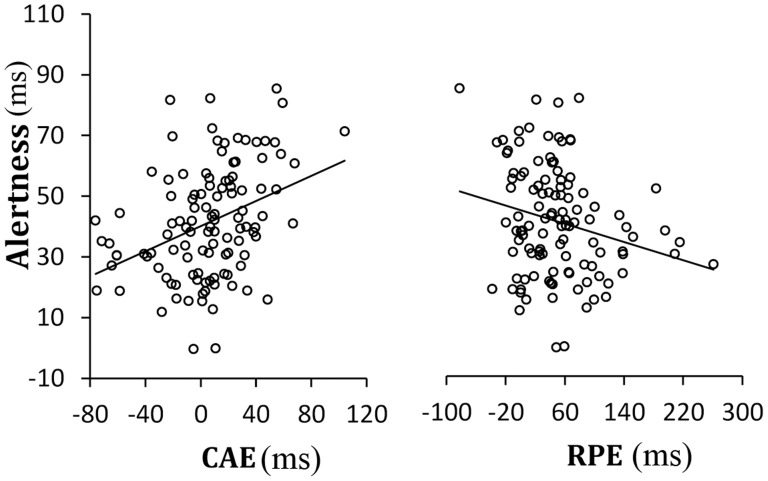
Scatter distribution in experiment 1. Curve fit between the efficiency of alertness and the CAE, alertness and the RPE, alertness and the congruency effect, respectively. NB. CAE  =  conflict adaptation effect on change trials; RPE  =  repetition priming effect on response or stimulus-response (S-R) repetition trials.

To establish a correlation between the attentional functions and the congruency sequence effect, we submitted all data for Pearson Correlation and bootstrapping correlation confidence interval to calculate for the correlation of interest. Results showed that the correlation between alerting and CAE, alerting and RPE, CAE and RPE, and executive control and conflict effect (the RTs difference of congruent and incongruent trials overall) was significant (the lower end of bootstrapping correlation confidence interval was above zero or the upper end was below zero, so the correlation was significant at the *p*<.05 level (two-tailed)). But the correlations between the orienting and CAE, orienting and RPE, executive control and CAE, and executive control and RPE were not significant (Table 1). Results of Pearson correlation were consistent with results of bootstrapping correlation. And the significant positive correlation between the conflict effect and Executive Control function of attentional network proves the reliability of the results of the experiment. Because the operational definition of the Executive Control function of attentional network was same to the Conflict effect. These results indicated that the alerting factor might significantly influence the congruency sequence effect (Figure 1), but both the orienting and control factors might not.

#### Individual differences based on the alerting level

To test the current hypothesis on whether the individual differences at the alerting level influenced the congruency sequence effect, we analyzed the correlations between the congruency sequence effect and attentional functions. Results showed that the correlation between alerting and congruency sequence effect was significant (Table 1). After the analysis, we divided the participants into two groups, the high alertness level group and the low alertness level group, using the median split of the alerting scores (range  = −1∼86 ms, *Median*  = 41 ms, *SD*  = 18).

To further examine the individual differences based on the alerting level, we used an independent samples t-test to examine the difference between the two groups. Results revealed that the alerting, the CAE, the RPE, and the error rates of the t-test between the two groups were significant, and that the orienting, the executive control, the conflict effect, and the mean RTs of the t-test between the two groups were not (Table 2). The overall mean RTs of the high alerting group was not different from that of the low alerting group, indicating that the intrinsic alertness between the two groups was not different. These results indicated that the efficiency of phasic alertness not only influenced the CAE, but also mediated the RPE and error rates (Table 2).

**Table 2:** Independent-samples t-test based on alerting level groups.

	t-test for Equality of Means				
	t	df	Sig.(2-tailed)	MD	SED
Alerting	14.655	110	.000	29.79	2.03
Orienting	.659	110	.511	2.58	3.92
EC	−.758	110	.450	−3.72	4.90
CAE	3.591	110	.000	21.22	5.91
RPE	−2.511	110	.013	−25.96	10.34
Conflict	−.362	110	.718	−1.23	3.41
Mean RT	.279	110	.781	3.31	11.88
Error	−2.521	110	.013	−.02	.01

NB. EC  =  executive control; CAE  =  conflict adaptation effect on change trials; RPE  =  repetition priming effect on the response or S-R repetition trials; conflict  =  congruency effects in the flanker task; RTs  =  reactive times in the task; and error  =  error rate in the task, respectively.

#### Repeated-measures ANOVAs for the congruency sequence effect on change trials and repetition trials

As done in previous studies, we conducted a contrast between (complete and partial) repetition and complete change trials for the overall performance. A three-way repeated-measures ANOVA (previous type × current type × transition type) was conducted. The three-way interaction was significant for both RTs (*F* (1, 111) = 44.35, *p*<.001, partial η^2^ = .285) and error rates (*F* (1, 111) = 10.03, *p*<.01, partial η^2^ = .082). To investigate the difference between repetition and change trials, two separate two-way repeated-measures ANOVAs were conducted on the critical interaction between previous and current types.

**Figure 2:**
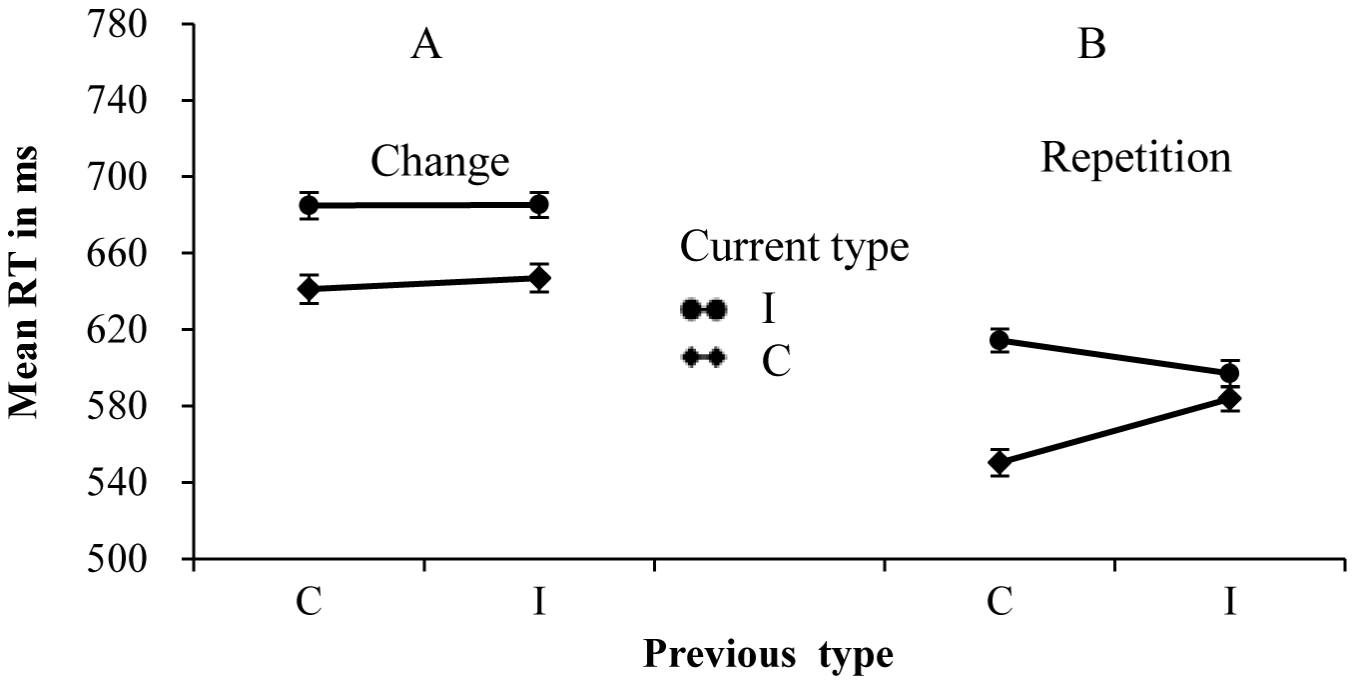
Mean RTs for each combination of current type and previous type. Panel A shows the results on change trials of all participants. Panel B shows the results on the response or S-R repetition trials of all participants.

On the change trials, the two-way interaction between current and previous types was marginally significant for RTs (*F* (1, 111) = 2.98, *p* = .09, partial η^2^ = .026), but was not significant for error rates (*F* (1, 111) = 1.12, *p* = .29, partial η^2^ = .010). The congruency effect (incongruent minus congruent) was 44 ms, following congruent trials, and 38 ms, following incongruent trials (Figure 2A). These results indicated that the CAE was marginally significant on the change trials. On the repetition trials, the two-way interaction was significant for both RTs (*F* (1, 111) = 91.85, *p*<.001, partial η^2^ = .453) and error rates (*F* (1, 111) = 15.21, *p*<.001, partial η^2^ = .121). The congruency effect was reduced from 64 ms, following congruent trials, to 13 ms, following incongruent trials (Figure 2B). These results indicated that the RPE was presented significantly on the response or S-R repetition trials.

If a large S-R set was used and the feature repetition was controlled, the CAE would be observed on change trials. The present results are consistent with previous studies [5], [33], in which the significant CAE was observed when the stimulus set of the flanker task was large. It was worth noting that the CAE was marginally significant on change trials in our studies. This may be caused by the individual difference in the alerting efficiency.

#### Repeated-measures ANOVAs for CAE and RPE

To confirm the influence of alertness on the two effects, we used a median split (41 ms) to divide the participants into a high-alertness group (whose alertness was higher than the median), and a low-alertness group (whose alertness was lower than the median). To investigate the differentiation of CAE and RPE between high-alertness and low-alertness groups, two separate three-way repeated-measures ANOVAs (previous type × current type × alerting level) were conducted.

**Figure 3:**
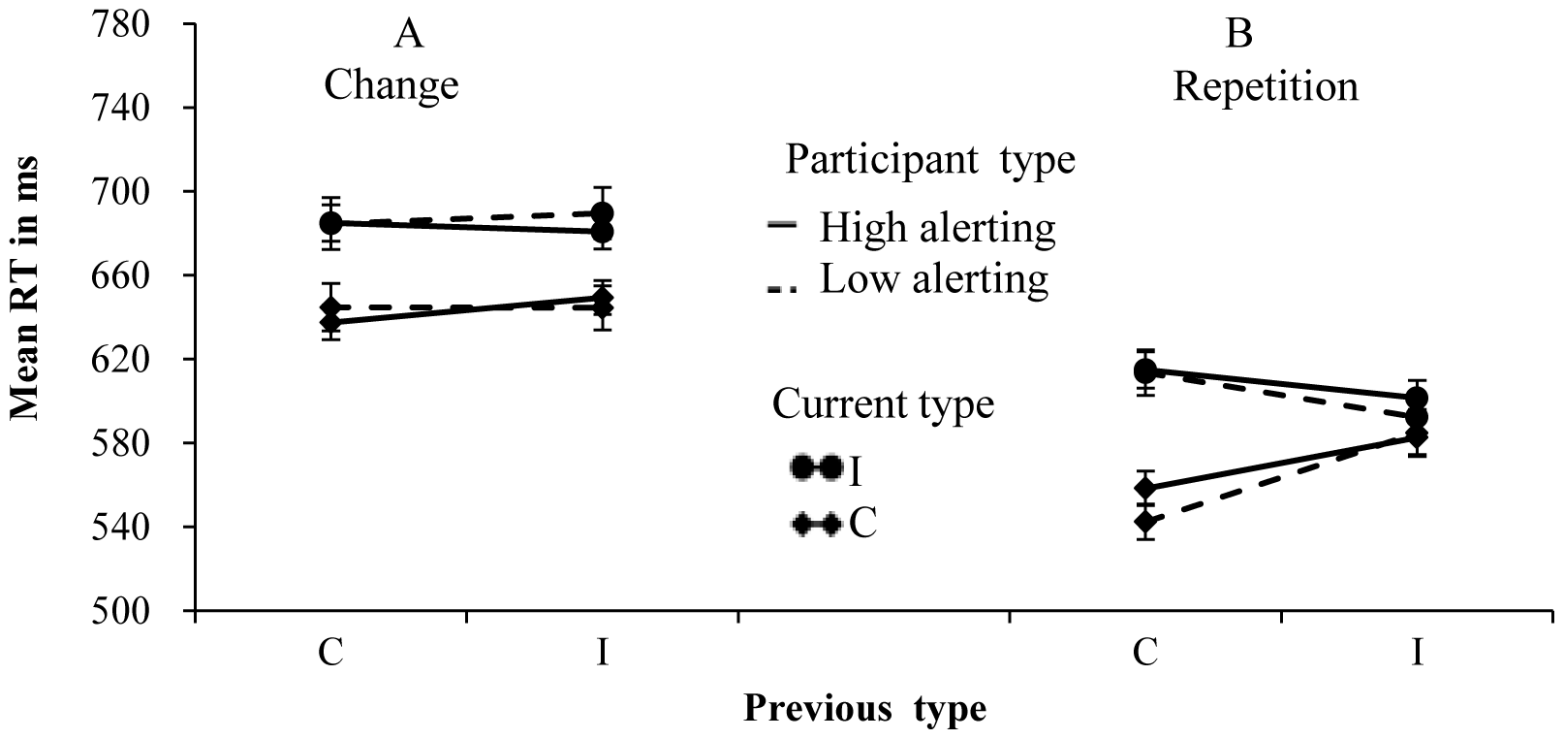
Mean RTs for each combination of current type and previous type in the two groups. Panel A shows the results on change trials of the high alerting level group and the low alerting level group. Panel B shows the results in the response or S-R repetition trials of the high alerting level group and the low alerting level group.

For the CAE, the previous type × current type × alerting level interaction was significant for RTs (*F* (1, 110) = 12.90, *p*<.001, partial η^2^ = .105), but not significant for error rates (*F* (1, 110) = 1.28, *p* = .26, partial η^2^ = .012). To investigate the differentiation between the high alertness group and the low alertness group, two separate two-way repeated-measures ANOVAs were conducted to explore the critical interaction between previous and current trial types. In the high alertness group, the two-way interaction between current and previous types was significant for RTs (*F* (1, 55) = 14.27, *p*<.001, partial η^2^ = .206), but not for error rates (*F* (1, 55)<1). The congruency effect was reduced from 47 ms, following congruent trials, to 32 ms, following incongruent trials (Figure 3A). In the low alertness group, the two-way interaction between current and previous types was not significant for both RTs (*F* (1, 55) = 1.16, *p* = .21, partial η^2^ = .028) and error rates (*F* (1, 55) = 2.46, *p* = .123, partial η^2^ = .043). The congruency effect was increased slightly from 40 ms, following congruent trials, to 45 ms, following incongruent trials (Figure 3A). Error rate data of different conditions were showed in Table 3. The results showed that the alertness did interact with the CAE and that the CAE was observed in the high-alertness group but not in the low-alertness group.

**Table 3:** Error rates (SE) for Transitions of Previous × Current Trial Type.

Type	Alertness	Trial n			
	Level	cC	cI	iC	iI
Change	High	.049(.005)	.064(.008)	.054(.006)	.068(.008)
	Low	.075(.008)	.087(.013)	.072(.010)	.101(.014)
Repetition	High	.021(.003)	.044(.006)	.032(.004)	.039(.005)
	Low	.036(.007)	.069(.010)	.060(.009)	.062(.010)

For the RPE, the interaction of previous × current type × alerting level was significant for RTs (*F* (1, 110) = 6.31, *p*<.05, partial η^2^ = .054), but not significant for error rates (*F* (1, 110) = 1.34, *p* = .25, partial η^2^ = .012). To investigate the differentiation between the high and low alertness groups, two separate two-way repeated-measures ANOVAs were conducted to explore the critical interaction between previous and current trial types. In the high alertness group, this interaction was significant for both RTs (*F* (1, 55) = 43.21, *p*<.001, partial η^2^ = .440) and error rates (*F* (1, 55) = 5.21, *p*<.05, partial η^2^ = .087). The congruency effect was reduced from 57 ms, following congruent trials, to 19 ms, following incongruent trials (Figure 3B). In the low alertness group, this interaction was significant for both RTs (*F* (1, 55) = 54.86, *p*<.001, partial η^2^ = .499) and error rates (*F* (1, 55) = 10.06, *p*<.01, partial η^2^ = .155). The congruency effect was reduced from 71 ms, following congruent trials, to 7 ms, following incongruent trials (Figure 3B). Error rate data of different conditions were showed in Table 3. These results indicated that alertness also interacts with the RPE.

Further analysis indicated that the CAE and RPE showed opposite patterns of effect in the two groups: the CAE of the high-alertness group was larger than that of the low-alertness group; however, the RPE of the high-alertness group was smaller than that of the low-alertness group (Table 2). Therefore, we concluded that the alertness functions influenced these two effects in opposing ways.

Interestingly, there was a significantly negative correlation between the CAE and RPE (Table 1), yet the sum of CAE and RPE were nearly equal across subject groups: 60 ms for all participants, 54 ms for the high-alertness participants, and 59 ms for the low-alertness participants. There was no significant difference in congruency effects between groups. This finding suggested that the two effects may have a competitive or complementary relationship.

## Experiment 2

In Experiment 1, the results indicated that the congruency sequence effect can result from both conflict monitoring and feature repetition, and the CAE and RPE may have a competitive or complementary relationship which was modulated by the alert functions of participants. However, the RPE may confound, to some extent, the influence of feature integration and conflict adaptation [10]. In addition, previous studies indicated that the FOE is uncontaminated by the CAE [10]. The FOE is calculated on iI trials which can constitute any of the four classes of feature overlap (complete repetition, complete change, distractor repetition only, and target repetition only) in the four-choice task [10]. To reveal the role of alertness in the congruency sequence effect in fine-grained details, we used the FOE, which does not contaminate CAE, in substitute for the RPE to stand for the feature integration effect in Experiment 2.

### Participants

A total of 70 right-handed undergraduate and graduate students (64 females; mean age  = 21 years, range  = 17−24) were paid for their participation. All participants had normal or corrected-to-normal vision. All participants provided written informed consent.

### Procedure and Design

The critical new manipulation in Experiment 2 was that there were 12 different types of incongruent trials (HHNHH, HHQHH, HHOHH, NNHNN, NNQNN, NNONN, OOHOO, OONOO, OOQOO, QQHQQ, QQNQQ, and QQOQQ), by which we can calculate the FOE. By contrast, there were only four types of incongruent trials in Experiment 1, limiting our ability to calculate the FOE. The other experimental parameters of Experiment 2 were the same as those used in Experiment 1.

### Results

Just like in Experiment 1, three attentional functions were calculated first. To evaluate the independence of attentional networks, we also submitted all data to Pearson Correlation and bootstrapping correlation confidence interval analysis among the three network effects. Results showed that the efficiencies of these three networks were uncorrelated (Table 4). Inasmuch as they had no correlation with one another, the scores of the three networks were used for further analysis.

**Table 4:** Correlations between the related factors in Experiment 2.

			Alerting	Orienting	EC	CAE	FOE
	Pearson Correlation		−.099	—			
**Orienting**	*Bootstrap^c^*	lower	−.310				
	*95% CI*	upper	.116				
	Pearson Correlation		.032	−.186	—		
**EC**	*Bootstrap^c^*	lower	−.229	−.391			
	*95% CI*	upper	.276	.043			
	Pearson Correlation		.289*	−.086	−.051	—	
**CAE**	*Bootstrap^c^*	lower	.070	−.324	−.249		
	*95% CI*	upper	.462	.170	.159		
	Pearson Correlation		−.048	−.078	−.039	−.089	—
**FOE**	*Bootstrap^c^*	lower	−.308	−.320	−.241	−.160	
	*95% CI*	upper	.243	.178	.177	.327	
	Pearson Correlation		.029	−.003	.292*	.214	.057
**Conflict**	*Bootstrap^c^*	lower	−.200	−.233	.068	−.044	−.199
	*95% CI*	upper	.245	.231	.480	.445	.319

* . Correlation is significant at the .05 level (2-tailed).

c. Unless otherwise noted, bootstrap results are based on 5000 bootstrap samples

NB. EC  =  executive control; CAE  =  conflict adaptation effect on change trials; FOE  =  feature overlap effect on the iI trials; and conflict  =  congruency effects in the flanker task; CI  =  Confidence Interval, respectively.

The preliminary analysis was the same as done in Experiment 1, We noted the RT_[(cI - cC) - (iI - iC)]_ for CAE. According to the prediction of feature integration account that the responses to partial repetitions will be slower than those to both complete changes and complete repetitions. So we used the processing difference between partial repetitions and complete repetitions or changes to stand for the feature integration effect. Therefore, we noted RT_[(iI2+iI3) - (iI1+iI4)]_ for FOE (iI1  =  complete repetition iI trials, iI2  =  only flanker repetition iI trials, iI3  =  only target repetition iI trials, and iI4  =  complete change iI trials, respectively).

**Figure 4:**
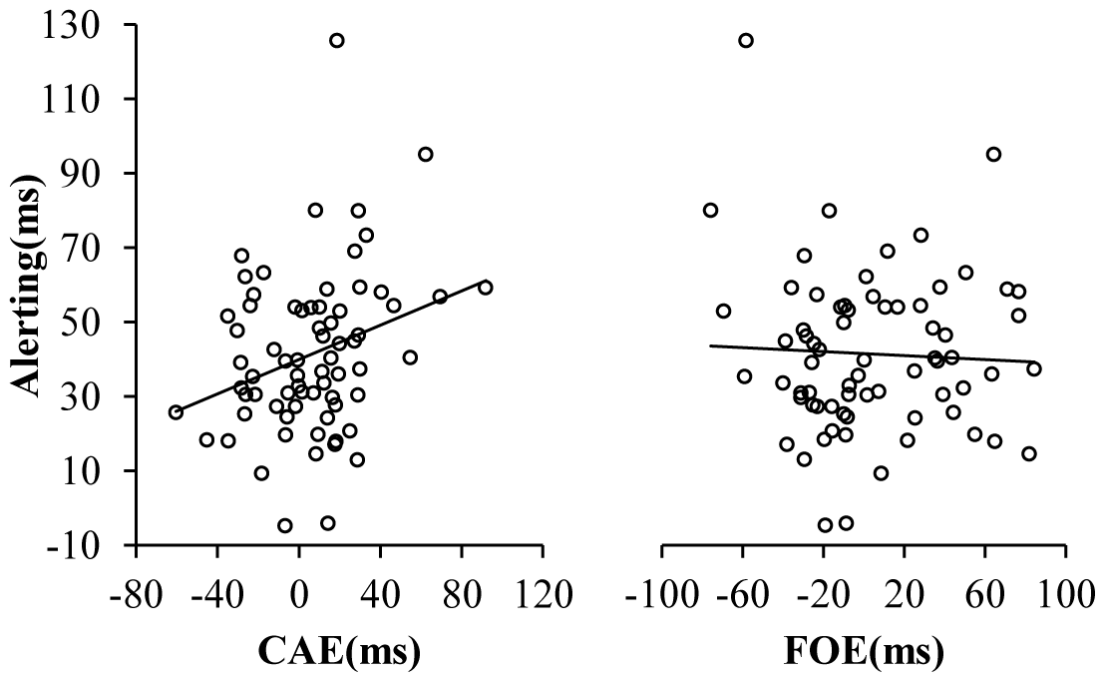
Scatter distribution in experiment 2. Curve fit between the efficiency of alertness and the CAE, alertness and the FOE, alertness and the congruency effect. NB. CAE  =  conflict adaptation effect on the change trials; FOE  =  feature overlap effect on the iI trials.

We also submitted all data for Pearson Correlation and bootstrapping correlation confidence interval to calculate for the correlation of interest. Results showed that the correlations between alerting and CAE, and executive control and conflict effect were significant (the lower end of bootstrapping correlation confidence interval was above zero or the upper end is below zero, so the correlation was significant at the *p*<.05 level (two-tailed)), whereas others were not (Table 4). Results of Pearson correlations were consistent with results of bootstrapping correlation. These results indicated that the alerting factor may significantly influence the congruency sequence effect (Figure 4), but the orienting and control factors may not.

#### Individual differences based on the alerting level

Consistent with Experiment 1, we divided the participants into two groups, the high alertness level group and the low alertness level group, using the median split of the alerting scores (41 ms).

Next, we used the independent samples *t*-test to examine the difference between the two groups. Results revealed that the alerting and the CAE by the *t*-test between the two groups were significant, but that the orienting, the executive control, the FOE, and the congruency effect by the *t*-test between the two groups were not (Table 5). These results indicated that the efficiency of phasic alertness only influenced the CAE, but not the FOE.

**Table 5:** Independent-samples t-test based on alerting level groups.

	t-test for Equality of Means				
	t	df	Sig.(2-tailed)	MD	SED
Alerting	9.654	68	.000	32.88	3.41
Orienting	−1.093	68	.278	−5.37	4.92
EC	−.312	68	.756	−1.63	5.21
CAE	2.54	68	.013	15.96	6.28
FOE	.180	68	.858	1.66	9.23
Conflict	.350	68	.727	1.59	4.54

NB. EC  =  executive control; CAE  =  conflict adaptation effect on change trials; FOE  =  feature overlap effect on the iI repetition trials; conflict  =  congruency effects in the flanker task.

#### Repeated-measures ANOVAs for the congruency sequence effect on both change trials and iI trials

**Figure 5:**
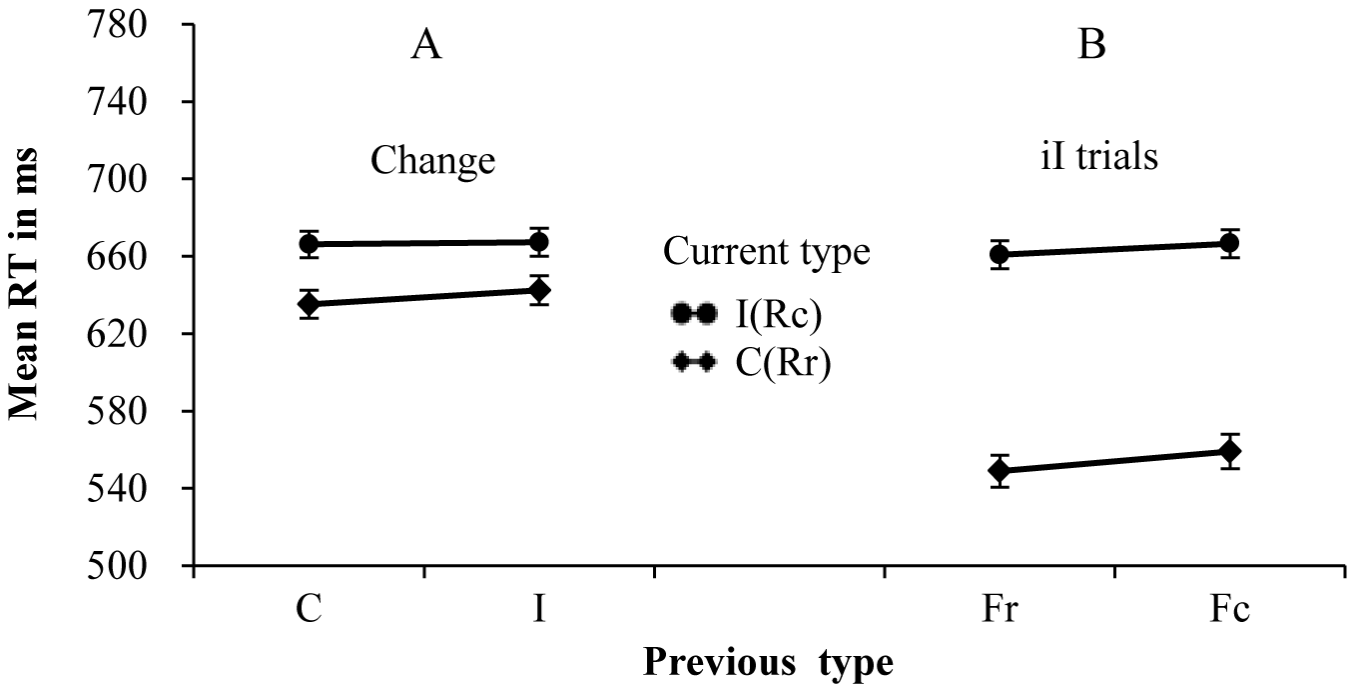
Mean RTs for each combination of current type and previous type. Panel A shows the results on change trials of all participants. Panel B shows the results on the iI trials of all participants. NB. I  =  incongruent trials, and C  =  congruent trials in panel A. Rc  =  response change, Rr  =  response repetition, Fr  =  flanker repetition, and Fc  =  flanker change in Panel B, respectively.

On the change trials, the two-way interaction between the current and the previous types was marginally significant for RTs (*F* (1, 69) = 3.55, *p* = .06, partial η^2^ = .049), but was not significant for error rates (*F* (1, 69)<1). The congruency effect was 31 ms, following congruent trials, and 25 ms, following incongruent trials (Figure 5A). These results indicated that the CAE was marginally significant in the change trials. On the iI trials, the two-way interaction was not significant in both RTs (*F* (1, 69)<1) (Figure 5B) and error rates (*F* (1, 69) = 1.17, *p* = .28, partial η^2^ = .017). These results indicated that the FOE was not presented on the iI trials.

#### Repeated-measures ANOVAs for the CAE and FOE

To investigate the differentiation of CAE and FOE between the high-alertness and low-alertness groups, two separate three-way repeated-measures ANOVAs were conducted: a three-way repeated-measures ANOVAs previous type (congruent/incongruent) × current type (congruent/incongruent) × alerting level for the CAE and another three-way repeated-measures ANOVAs flanker type (repetition/change) × response type (repetition/change) × alerting level for the FOE.

**Figure 6:**
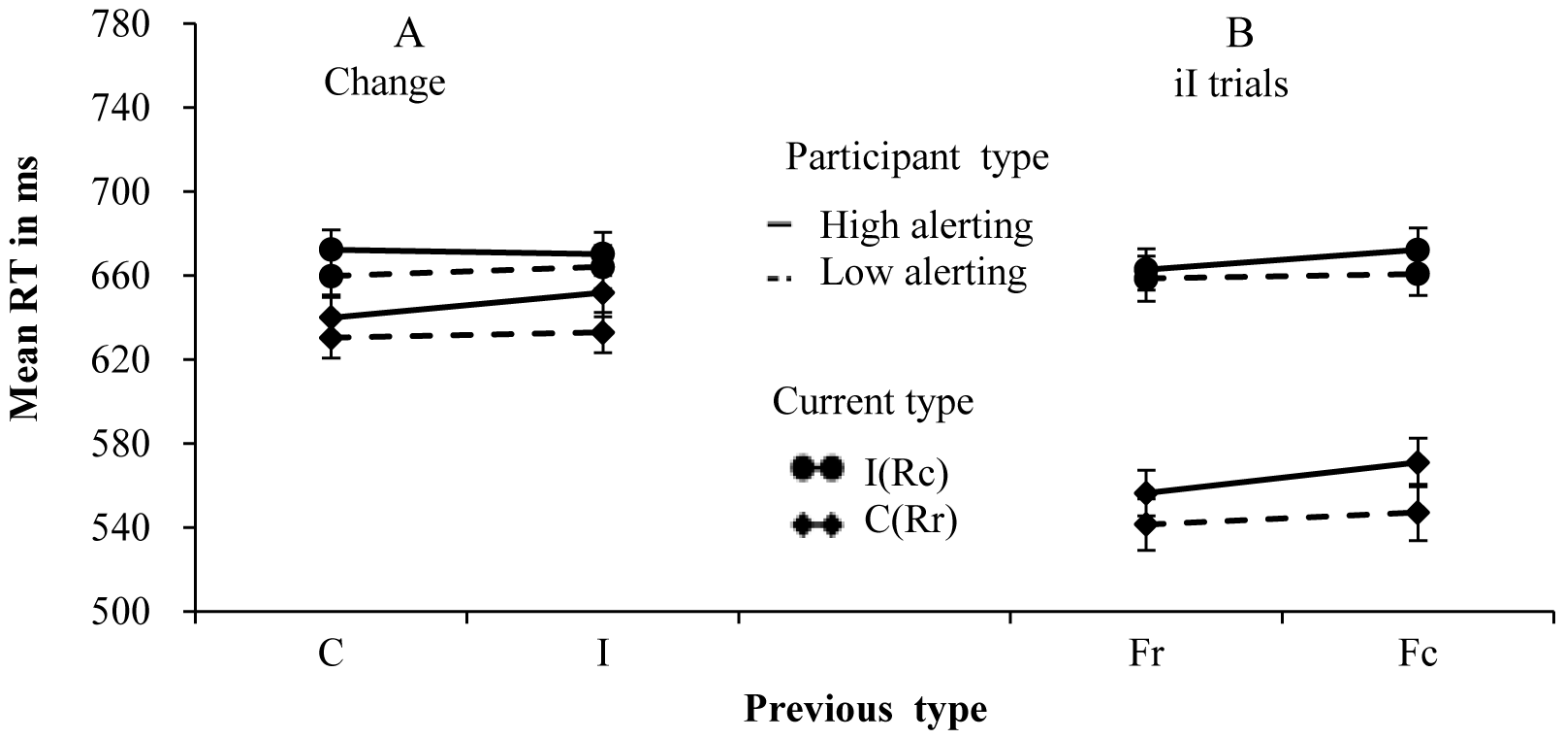
Mean RTs for each combination of current type and previous type of the two groups. Panel A shows the results on change trials of the high alerting level group and the low alerting level group. Panel B shows the results on the iI trials of the high alerting level group and the low alerting level group, respectively. NB. I  =  incongruent trials, C  =  congruent trials in panel A. Rc  =  response change, Rr  =  response repetition, Fr  =  flanker repetition, and Fc  =  flanker change in Panel B, respectively.

For the CAE, the interaction of previous type × current type × alerting level was significant for RTs (*F* (1, 68) = 6.46, *p*<.05, partial η^2^ = .087) but not for error rates (*F* (1, 68) = 2.44, *p* = .12, partial η^2^ = .035). To investigate the differentiation between the high alertness group and low alertness group, two separate two-way repeated-measures ANOVAs were conducted to explore the critical interaction between previous and current trial types. In the high alertness group, the two-way interaction between current and previous types was significant for RTs (*F* (1, 34) = 7.96, *p*<.01, partial η^2^ = .19), but not for error rates (*F* (1, 34)<1). The congruency effect was reduced from 33 ms, following congruent trials, to 19 ms, following incongruent trials (Figure 6A). In the low alertness group, the two-way interaction between current and previous types was not significant for both RTs (*F* (1, 34)<1) and error rates (*F* (1, 34) = 2.63, *p* = .114, partial η^2^ = .072). The congruency effect was increased slightly from 29 ms, following congruent trials, to 32 ms, following incongruent trials (Figure 6A). Error rate data of different conditions are showed in Table 6. The results showed that the alertness interacted with the CAE and indicated that the CAE was observed in the high-alertness group but not in the low-alertness group.

**Table 6:** Error rates (SE) for Transitions of Previous × Current Trial Type in experiment 2.

Type	Alertness	Trial n			
	Level	cC	cI	iC	iI
Change	High	.052(.005)	.070(.008)	.049(.007)	.072(.015)
	Low	.046(.008)	.064(.009)	.049(.009)	.055(.012)
		iI1	iI2	iI3	iI4
iI	High	.020(.005)	.064(.009)	.021(.005)	.067(.011)
	Low	.024(.006)	.076(.012)	.024(.005)	.056(.011)

NB. iI1  =  complete repetition iI trials; iI2  =  only flanker repetition iI trials; iI3  =  only response repetition iI trials, and iI4  =  complete change iI trials, respectively.

For the FOE, the interaction of flanker type (repetition/change) × response type (repetition/change) × alerting level was not significant for RTs (*F* (1, 68)<1), and error rates (*F* (1, 68) = 1.47, *p* = 0.229, partial η^2^ = .021). To investigate the differentiation between the high and low alertness groups, two separate two-way repeated-measures ANOVAs were conducted to explore the critical interaction between flanker and response types. In the high alertness group, this interaction was not significant for both RTs (*F* (1, 34)<1) (Figure 6B) and error rates (*F* (1, 34)<1). In the low alertness group, this interaction was not significant for both RTs (*F* (1, 34)<1) (Figure 6B) and error rates (*F* (1, 34) = 3.31, *p* = .08, partial η^2^ = .089). Error rate data of different conditions are showed in Table 6. These results indicated that alertness did not interact with the FOE and the FOE was not consistent with the prediction made by the feature integration account.

In Experiment 2, the results indicated that the alertness only affected the CAE, but not the FOE. We also calculated the RPE as done in Experiment 1, and the results was similar to those found in Experiment 1: there was also a significant negative correlation between alertness and RPE (*r* = -.255, *p*<.05), and a marginally significant negative correlation between CAE and RPE (*r* = −.213, *p* = .077) in Experiment 2. Although the RPE may confound the feature sequences with CAE, the negative correlation between CAE and RPE was observed in both experiments.

## General Discussion

In two experiments, we found a significant positive correlation between the alertness and the CAE, and a negative correlation between the alertness and the RPE. Moreover, a significant negative correlation existed between CAE and RPE in Experiment 1, and a marginally significant negative correlation existed between CAE and RPE in Experiment 2. However, in Experiment 2, the correlation between alertness and FOE, and the correlation between CAE and FOE were not significant. And the FOE was not consistent with the prediction made by the feature integration account. The feature integration account predicts that responses to partial repetitions will be slower than those to both complete changes and complete repetitions. The present results for the FOE were similar to those of Ackay and Hazeltine who found that the partial repetitions did not lead to slower performance than the complete-change sequences, and RTs on complete-change trials were significantly longer than those on location repetition/response change trials when any confounding effect of correspondence and negative priming was removed, and they suggested that the FOE was accounted better by strategic shortcuts in response selection [10]. Thus, the mechanism of RPE on response or S-R repetition trials was different from the FOE on iI trials, and it was inappropriate to use results of FOE-related to infer the relationship between the alertness and feature integration effect.

Although the RPE may confound, to some extent, the influence of feature integration and conflict adaptation, it was more appropriate to stand for the feature integration effect than FOE that was calculated on iI trials. Theoretically, the CAE in the change trials should have a positive correlation with the CAE in response or S-R repetition trials. If there were a significant negative correlation between CAE and RPE (RPE here contained CAE on response or S-R repetition trials), mathematically, there would be a more significant negative correlation between CAE and “pure” RPE (RPE here contained no CAE). Therefore, the results of the relations between alertness and RPE, CAE and RPE in Experiments 1 and 2 should be credible. Also, the results of RPE-related in Experiments 1 and 2 suggest that there may be a real negative correlation between alertness and feature integration effect. Thus, the focus of the present study was why there were the significant correlations between alertness and the congruency sequence effect which contained CAE and RPE, and how the alertness mediates the congruency sequence effect.

At the neural level, evidence from neuroimaging studies had identified neural correlates of congruency sequence effect to be predominant in the medial prefrontal cortex, particularly in the ACC [25]. Verguts & Notebaert [26] proposed that the ACC exerted its effect on DLPFC indirectly through LC-related processing in the congruency sequence effect. Meantime, both the ACC and the LC–norepinephrine (NE) system are involved in alertness-related modulation[34], [35]. Therefore, the significant correlation between alertness and the congruency sequence effect may be due to that they share common processes with the LC-ACC loop mechanisms[34], [35].

In the current study, there was a significant positive correlation between alertness and CAE and a significant negative correlation between alertness and RPE. Participants at the high alerting level group had a larger CAE and a smaller RPE than participants at the low alerting level group. These results indicated the alertness may modulate the conflict adaptation and feature integration with an opposite way. In sequential modulations, participants at the high alerting level group performed better on the change trials, whereas participants at the low alerting level performed better on the response or S-R repetition trials. A possible interpretation about this phenomenon is that different control strategies may be employed by the participants of these two groups. Specifically, participants at the high alerting level group tend to use the top-down cognitive processing strategy, whereas participants at the low alerting level group tend to use the bottom-up processing strategy. This inference is supported by the dual mechanism of cognitive control [36], which proposed that the high alerting participants show a proactive control performance pattern, whereas the low alerting participants show a reactive control performance pattern. The proactive control mode refers to a goal-driven manner, which is actively maintained in a sustained manner before the occurrence of cognitively demanding events. In the reactive mode, the attentional control is employed as a “late correction” mechanism that is mobilized only as needed in an event-driven manner. Thus, participants at different alerting levels may take different cognitive control processing strategies. In addition, there was also a significant negative correlation between CAE and RPE. This finding prompted that CAE and RPE may have a competitive or complementary relationship and this relationship was worth to do more researches in the future.

In the past, Mayr et al. [7], Nieuwenhuis et al. [2], and Wendt et al. [37] found that, after excluding the S-R repetitions, the CAE was not present. This result is different from our findings. In their studies, the individual differences in the alerting level may impair the underlying the CAE. However, Ullsperger et al. [5], Verbruggen et al. [33], and Notebaert & Verguts [38] observed a significant CAE after controlling the feature integration effect. Ullsperger et al. [5] used a speeded task, in which a feedback was provided on each trial, indicating that participants should speed up their responses. Other experimental manipulations, such as a warning tone followed by a preparatory period, could also be used to increase the alertness of participants. Evidence from previous studies suggested that an increased alertness level can be indexed by a more rapid response to subsequent events [27], [39]. Yanaka et al. [17] proposed that a cue presentation enhances alertness phasically, which, in turn, facilitates the preparation of a motor response. Verbruggen et al. used a modified flanker task, in which six color stimulus values were mapped onto three response buttons, and a feedback was used to accelerate the response on each trial. Notebaert et al. used a numerical flanker task, where the target-flanker distance could be changed, and found an adaptation was triggered by high levels of stimulus conflict, which might be detected easily even if the alert level was low.

An interesting finding was that the correlation between the alerting level and conflict effect was not significant, whereas the correlation between the alerting level and the CAE was. This result suggests that the conflict control and control adaptation may have different mechanisms in the brain. Previous studies have also shown that the cognitive control encompasses two separate forms, namely, the conflict control and the control adaptation [40], [41].

In conclusion, the present study reveals that alertness can modulate conflict adaptation and feature integration in an opposite way. In sequential modulations, participants at the high alerting level group performed better on change trials, whereas participants at the low alerting level performed better on response or S-R repetition trials. A possible interpretation about this phenomenon is that participants at the high alerting level group may tend to use the top-down cognitive processing strategy, whereas participants at the low alerting level group tend to use the bottom-up processing strategy.

## Supporting Information

Table S1 The stimuli arrays and transitions used in the flanker task. Notes: The trial sequences of “re/ch” column are the following: 1 is complete repetition, 2 is partial repetition, and 3 is complete change. The one flanker letter here represents two flanker letters in real experiment. To control the feature negative priming effect on change trials, the trials in bold and italic type are not contained in the flanker task.
(DOC)
