# Supplementary material for: Alertness Modulates Conflict Adaptation and Feature Integration in an Opposite Way
Source: PLoS One. 2013 Nov 8;8(11):e79146. doi: 10.1371/journal.pone.0079146 (PMC3826738; doi:10.1371/journal.pone.0079146)
Supplement: Table S1 — The stimuli arrays and transitions used in the flanker task. Notes: The trial sequences of “re/ch” column are the following: 1 is complete repetition, 2 is partial repetition, and 3 is complete change. The one flanker letter here represents two flanker letters in real experiment. To control the feature negative priming effect on change trials, the trials in bold and italic type are not contained in the flanker task. (DOC) [file pone.0079146.s001.doc]

**Supporting Information**

**Table S1.** The stimuli arrays and transitions used in the flanker task. Notes: The trial sequences of “re/ch” column are the following: 1 is complete repetition, 2 is partial repetition, and 3 is complete change. The one flanker letter here represents two flanker letters in real experiment. To control the feature negative priming effect on change trials, the trials in bold and italic type are not contained in the flanker task.

| CC | | | II | | | CI | | | IC | | |
| --- | --- | --- | --- | --- | --- | --- | --- | --- | --- | --- | --- |
| trial*_n-1_* | trial*_n_* | re/ch | trial*_n-1_* | trial*_n_* | re/ch | trial*_n-1_* | trial*_n_* | re/ch | trial*_n-1_* | trial*_n_* | re/ch |
| HHH | HHH | 1 | HNH | HNH | 1 | ***HHH*** | ***HNH*** | ***2*** | ***HNH*** | ***HHH*** | ***2*** |
| NNN | NNN | 1 | NHN | NHN | 1 | HHH | NHN | 2 | HNH | NNN | 2 |
| OOO | OOO | 1 | OQO | OQO | 1 | NNN | HNH | 2 | NHN | HHH | 2 |
| QQQ | QQQ | 1 | QOQ | QOQ | 1 | ***NNN*** | ***NHN*** | ***2*** | ***NHN*** | ***NNN*** | ***2*** |
| ***HHH*** | ***NNN*** | ***2*** | ***HNH*** | ***NHN*** | ***2*** | ***OOO*** | ***OQO*** | ***2*** | ***OQO*** | ***OOO*** | ***2*** |
| HHH | OOO | 3 | ***NHN*** | ***HNH*** | ***2*** | OOO | QOQ | 2 | OQO | QQQ | 2 |
| HHH | QQQ | 3 | ***OQO*** | ***QOQ*** | ***2*** | QQQ | OQO | 2 | QOQ | OOO | 2 |
| ***NNN*** | ***HHH*** | ***2*** | ***QOQ*** | ***OQO*** | ***2*** | ***QQQ*** | ***QOQ*** | ***2*** | ***QOQ*** | ***QQQ*** | ***2*** |
| NNN | OOO | 3 | HNH | OQO | 3 | HHH | OQO | 3 | HNH | OOO | 3 |
| NNN | QQQ | 3 | HNH | QOQ | 3 | HHH | QOQ | 3 | HNH | QQQ | 3 |
| OOO | HHH | 3 | NHN | OQO | 3 | NNN | OQO | 3 | NHN | OOO | 3 |
| OOO | NNN | 3 | NHN | QOQ | 3 | NNN | QOQ | 3 | NHN | QQQ | 3 |
| ***OOO*** | ***QQQ*** | ***2*** | OQO | HNH | 3 | OOO | HNH | 3 | OQO | HHH | 3 |
| QQQ | HHH | 3 | OQO | NHN | 3 | OOO | NHN | 3 | OQO | NNN | 3 |
| QQQ | NNN | 3 | QOQ | HNH | 3 | QQQ | HNH | 3 | QOQ | HHH | 3 |
| ***QQQ*** | ***OOO*** | ***2*** | QOQ | NHN | 3 | QQQ | NHN | 3 | QOQ | NNN | 3 |
